# The independent and combined effects of floral traits distinguishing two pollination ecotypes of a moth‐pollinated orchid

**DOI:** 10.1002/ece3.4808

**Published:** 2019-01-23

**Authors:** Judith Trunschke, Nina Sletvold, Jon Ågren

**Affiliations:** ^1^ Plant Ecology and Evolution, Department of Ecology and Genetics, Evolutionary Biology Centre Uppsala University Uppsala Sweden

**Keywords:** correlated traits, correlational selection, local adaptation, phenotypic manipulation, pollination efficiency, pollinator attraction, pollinator‐mediated selection

## Abstract

Identifying traits and agents of selection involved in local adaptation is important for understanding population divergence. In southern Sweden, the moth‐pollinated orchid *Platanthera bifolia* occurs as a woodland and a grassland ecotype that differ in dominating pollinators. The woodland ecotype is taller (expected to influence pollinator attraction) and produces flowers with longer spurs (expected to influence efficiency of pollen transfer) compared to the grassland ecotype. We examined whether plant height and spur length affect pollination and reproductive success in a woodland population, and whether effects are non‐additive, as expected for traits influencing two multiplicative components of pollen transfer. We reduced plant height and spur length to match trait values observed in the grassland ecotype and determined the effects on pollen removal, pollen receipt, and fruit production. In addition, to examine the effects of naturally occurring variation, we quantified pollinator‐mediated selection through pollen removal and seed production in the same population. Reductions of plant height and spur length decreased pollen removal, number of flowers receiving pollen, mean pollen receipt per pollinated flower, and fruit production per plant, but no significant interaction effect was detected. The selection analysis demonstrated pollinator‐mediated selection for taller plants via female fitness. However, there was no current selection mediated by pollinators on spur length, and pollen removal was not related to plant height or spur length. The results show that, although both traits are important for pollination success and female fitness in the woodland habitat, only plant height was sufficiently variable in the study population for current pollinator‐mediated selection to be detected. More generally, the results illustrate how a combination of experimental approaches can be used to identify both traits and agents of selection.

## 1 INTRODUCTION

Among‐population variation in interactions with pollinators may result in divergent selection on floral traits, promoting floral diversification (Grant, 1949, 1994; Stebbins, 1970; Van der Niet & Johnson, 2012; Van der Niet, Peakall, & Johnson, 2014), and the formation of pollination ecotypes (Anderson, Alexandersson, & Johnson, 2010; Johnson, 1997; Van der Niet, Pirie, Shuttleworth, Johnson, & Midgley, 2014). However, ecotypes (e.g., Armbruster, 1985; Boberg et al., 2014; Van der Niet, Pirie, et al., 2014) and closely related species (e.g., Campbell, Waser, & Melendez‐Ackerman, 1997; Sun, Schlüter, Gross, & Schiestl, 2015) commonly differ in multiple traits that can be strongly correlated within and across populations, and the relative contribution of different traits to adaptive differentiation is usually poorly known. Because of strong trait correlations, experimental approaches are required to determine the independent and combined effects of individual traits on pollination success and plant fitness (Campbell, 2009; Castellanos, Wilson, & Thomson, 2004; Schemske & Bradshaw, 2008).

The effects of two traits on fitness can be additive, such that they influence fitness independently, or non‐additive, such that the effect of one trait depends on the expression of the other. For example, traits enhancing pollinator visitation and those improving efficiency of pollen transfer should affect pollen removal and pollen receipt non‐additively because total pollen transfer is the product of number of pollinator visits and mean amount of pollen transferred per visit (Sletvold & Ågren, 2011). In pollen‐limited populations with sufficiently large trait variation, such non‐additive effects should be reflected as correlational selection. Yet, few studies have experimentally tested for non‐additive effects of floral characters affecting attraction of pollinators and those affecting efficiency of pollen transfer (Boberg & Ågren, 2009; Fenster, Cheely, Dudash, & Reynolds, 2006; Fenster, Reynolds, Williams, Makowsky, & Dudash, 2015; Sletvold & Ågren, 2011), and the prevalence of correlational selection on floral traits is largely unknown (but see e.g., Maad, 2000; Chapurlat, Ågren, & Sletvold, 2015; Toräng et al., 2017).

In hermaphroditic species, pollinator‐mediated selection through components of male and female fitness may differ for several reasons. First, the effect of a given floral character on pollen removal and pollen receipt may differ in both magnitude and direction (Delph & Ashman, 2006; Ellis & Johnson, 2010). Second, opportunity for selection (the variance in relative fitness; Crow, 1958) may differ between male and female reproductive success. Bateman’s principle predicts that the variance in siring success will be higher than the variance in female reproductive success because of differences in the relative importance of mate and resource limitation for male and female function (Bateman, 1948). However, not all components of male reproductive success are likely to display greater variance than does female fitness. For example, pollen removal may be considerably less variable than pollen receipt and fruit set, particularly if pollen is packed into units, such as orchid pollinia, facilitating efficient pollen transfer to flower visitors (Johnson & Edwards, 2000; Johnson, Neal, & Harder, 2005). The few available comparisons of opportunity for selection through male and female reproductive success in plants suggest that the direction of the difference varies (Delph & Ashman, 2006; and references therein), but differences in the approach used to estimate male reproductive success complicates comparisons across studies (Ashman & Morgan, 2004). More specifically, because pollen removal and male reproductive success may be weakly correlated (Johnson et al., 2005; Snow & Lewis, 1993), estimates of opportunity for selection through pollen removal may not reflect opportunity through male reproductive success. Understanding the effect of trait variation on pollen removal is still of interest as pollen removal is an important aspect of pollen transfer, and can be considered one component of male reproductive success.

In southern Sweden*,* plant stature and floral morphology differ between two distinct ecotypes of the moth‐pollinated orchid *Platanthera bifolia*. The woodland ecotype begins to flower about 2 weeks earlier and produces taller inflorescences, and more and larger flowers with longer spurs compared to the grassland ecotype (Boberg et al., 2014). A reciprocal transplant experiment indicated that morphological differences have a genetic basis (Boberg & Ågren, 2009). Reduced pollen removal and female fitness following shortening of spurs (Boberg & Ågren, 2009; Nilsson, 1988) suggest that the difference in spur length is important for the reproductive success of the woodland ecotype in its home environment, whereas the adaptive significance of the difference in plant height has not been examined. Plant height can be expected to be important for shading avoidance and efficient light capture but should also influence long‐distance attraction and the number of pollinator visits (Donnelly, Lortie, & Aarssen, 1998; Dudash, Hassler, Stevens, & Fenster, 2011; Engel & Irwin, 2003; Walsh, Arnold, & Michaels, 2014). Moreover, because spur length should influence the efficiency of pollen transfer at each visit (Muchhala & Thomson, 2009; Nilsson, 1988), non‐additive effects of these two traits on pollen transfer and female reproductive success can be expected (Sletvold & Ågren, 2011).

Here, we examine the independent and combined effects of plant height and spur length on pollen removal and measures of female reproductive success in a woodland population of *P. bifolia* using two approaches. First, we manipulated plant height and spur length to match trait values previously documented in the grassland ecotype and examined the effects on pollen removal, pollen receipt, and fruit production in a field experiment. Second, we examined the effects of naturally occurring trait variation in the woodland population by quantifying pollinator‐mediated selection through pollen removal and female fitness on plant height, spur length, and two additional traits potentially influencing attractiveness to pollinators (number of flowers and individual flower size). Pollinator‐mediated selection through female fitness was quantified by comparing estimates of selection among plants receiving supplemental hand‐pollination (non‐pollinator‐mediated selection) and estimates of selection among open‐pollinated plants (net selection; Sandring & Ågren, 2009; Sletvold & Ågren, 2010). Specifically, we tested the predictions that: (a) Reductions in plant height and spur length to those observed in the grassland ecotype reduce pollen removal, pollen receipt, and fruit production in the woodland population, (b) Effects of plant height and spur length on pollen transfer are non‐additive, resulting in pollinator‐mediated correlational selection, and (c) The opportunity for selection is higher and pollinator‐mediated selection is stronger through female fitness than through pollen removal.

## 2 MATERIAL AND METHODS

### 2.1 *Platanthera bifolia* and its ecotypes

**Figure 1:**
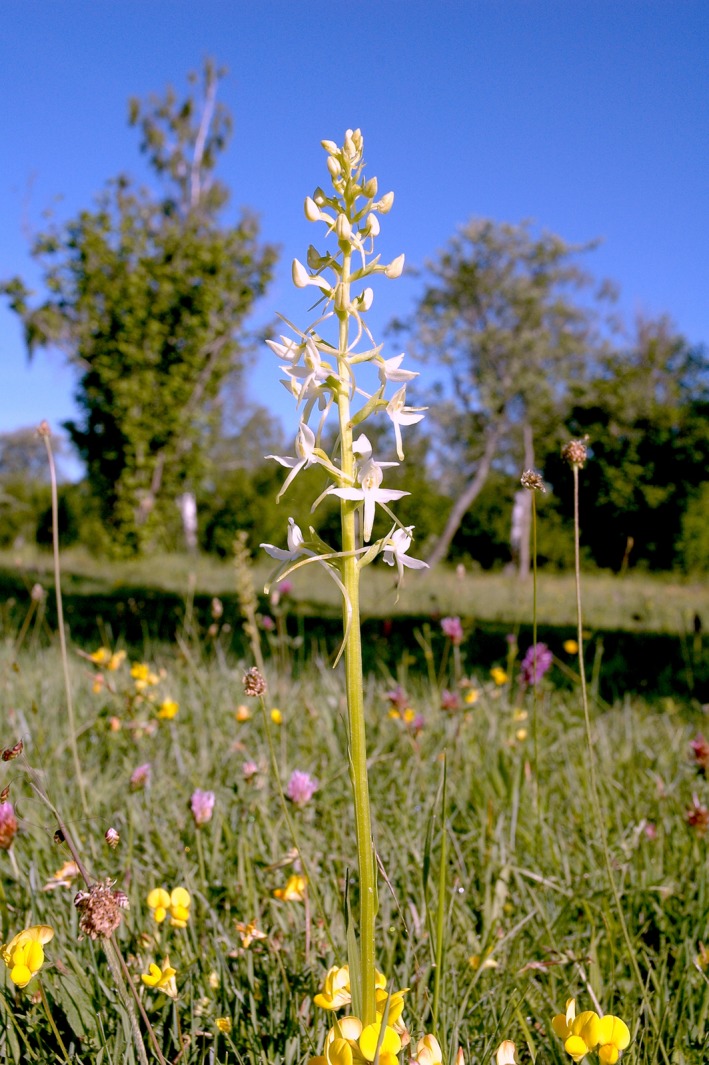
*Platanthera bifolia* on the island Öland, SE Sweden. Photo: Judith Trunschke

*Platanthera bifolia* (L.) Rich. is a long‐lived terrestrial orchid that is widespread in the temperate regions across Eurasia (Delforge, 2005). The species is found in a wide range of habitats including grasslands, moorlands, marshes, and woodlands, and occurs at elevations up to 2,500 m asl. Flowering starts in early‐ to mid‐June in southern Sweden and individual plants produce a single inflorescence in which flowers open sequentially from bottom to top within a few days (Figure 1). Flowers are white, and at night emit a strong scent that is dominated by benzenoids and linalool (Tollsten & Bergström, 1989, 1993). Sugar‐rich nectar is secreted from unicellular hairs that cover the inside walls of the spur (Stpiczynska, 1997). The species is nocturnally pollinated and attracts a variety of sphingid and noctuid moths (Boberg et al., 2014; Claessens, Gravendeel, & Kleynen, 2008; Nilsson, 1983). Pollen is organized in massulae, which are packaged into two sectile pollinia per flower (hundreds of massulae per pollinium). Pollen from a given pollinium can be deposited in several flowers.

On the island Öland, southern Sweden, *P. bifolia* occurs as two morphologically distinct ecotypes in woodlands and grasslands, respectively (Boberg et al., 2014). The two ecotypes differ in flowering phenology, plant height, and floral traits (Boberg & Ågren, 2009; Boberg et al., 2014), but do not differ in the composition of the floral scent (Tollsten & Bergström, 1993). The woodland ecotype is usually found on deep moraine and clay soils with relatively tall ground vegetation, whereas the grassland ecotype is found on thin, nutrient‐poor sandy soils with short ground vegetation. On Öland, woodland populations are predominantly pollinated by the long‐proboscis hawkmoth *Sphinx ligustri* (proboscis length mean ± *SE*, 39.1 ± 2.2 mm, *N* = 52; data from Boberg et al., 2014), whereas grassland populations are pollinated by various moths with markedly shorter proboscis (*Deilephila porcellus* 17.9 ± 1.0 mm, *N* = 22 [Boberg et al., 2014], *Hyles gallii* 25.13 ± 0.49 mm, *N* = 2, and *Cucullia umbratica* 19.47 ± 0.06 mm, *N* = 11; measurements of specimens collected in *P. bifolia* populations on Öland). For the present study, we selected a large woodland population in the central part of Öland (Gråborg; N56°40′, E16°35′).

### 2.2 Experimental trait manipulation

To examine the effects of reduced plant height and spur length on pollination success and fruit production in the woodland ecotype, we conducted a field experiment with four treatments: (1) tall plants with long spurs (unmanipulated control), (2) tall plants with shortened spurs, (3) shortened plants with long spurs, and (4) shortened plants with shortened spurs. Phenotypic manipulations (plant height, 38 cm vs. 27 cm; spur length, 36 mm vs. 21 mm) corresponded to the respective mean trait values previously documented in the study population (long‐spurred) and in a nearby population of the grassland ecotype (short‐spurred; Melösa, N56°51′, E16°51′; Boberg et al., 2014). In the year of the field experiment, plants in the Gråborg population were somewhat taller (mean ± *SE*, 42.8 ± 1.3 cm) and produced somewhat longer spurs (38.5 ± 0.8 mm; *N* = 23) compared to the previous year.

In early June 2015, we marked close to 200 bolting plants and bagged them to prevent pollinator visitation before the start of the experiment. Once flowering, we recorded plant height as the distance from the ground to the uppermost flower of the inflorescence to the nearest 0.1 cm with a ruler, counted the number of flowers, and measured corolla height and width (vertical and horizontal distance between petal tips), and spur length (from entrance to tip) to the nearest 0.01 mm on the second fully expanded flower of each experimental plant. Flower size was quantified as the product of corolla height and width.

The experiment was staggered over a 2‐week period in mid‐June 2015. On each of five experimental days, we established three to six blocks, for a total of 24 blocks. Each block contained four plants (one plant of each of the four treatments). Any senescent flowers and/or buds were removed prior to the experiment. Plants were assigned to blocks such that all plants in a block had the same number of open flowers (mean [range] number of open flowers, 18 [12–23], *N* = 24 blocks). Within a block, the four treatments were randomly assigned with the exception that plants in treatment three (shortened plants with long spurs) had to have spurs that were at least 36 mm long. If a given plant did not meet this criterion, a new plant with the same number of open flowers was selected among plants bagged for the experiment. Experimental plants were cut above the basal leaves and were kept in floristic vials filled with water and added orchid liquid fertilizer (BAYER Garden, NPK 2:1:1).

Spur manipulations were conducted in the lab at the nearby biological field station in Skogsby (Station Linné). Each spur on an inflorescence was manipulated by carefully squeezing the nectar from the tip upwards, and then gently folding and fixing the spur tip with a thin piece of green elastic tape to obtain a length of either 36 or 21 mm (Figure 1a). In the control treatment (unmanipulated tall plants and long spurs), we attached a small piece of tape to each spur to control for effects of tape application. After spur manipulations, all inflorescences were returned to the field, and the vials were mounted on bamboo sticks placed besides the remaining basal leaves in each original plant position. Inflorescence height was manipulated by adjusting the height of the vial, such that the top flower was positioned at either 38 or 27 cm (Figure 1b) above the ground. The height of inflorescences in the control treatment was adjusted to their original height prior to cutting.

In the field, experimental inflorescences were exposed to pollinators for three consecutive nights and after each night the number of pollinia removed and the number of massulae received was scored for all flowers (see Supporting Information Figure S1). Total number of pollinia removed per plant was calculated based on observations after the third night, the number of flowers receiving pollen during the experiment was noted, and cumulative pollen receipt per pollinated flower was quantified as the sum of the number of new massulae recorded on stigmas after each of the three nights. After the third night, all inflorescences were brought to the field station, where they were kept outdoors, and covered by a net cage to prevent further pollination. Inflorescences were provided with fertilized water on a weekly basis, and the number of fruits produced was scored after 4 weeks when fruits were maturing.

Two plants were excluded from the analysis of pollen removal and receipt due to damage by slugs during flowering, and 11 plants were excluded from the analysis of fruit production due to withering during fruit maturation. In addition, we excluded from estimates of pollen receipt per pollinated flower observations where entire pollinia were found on stigmas due to the uncertainty regarding the number of massulae in contact with the stigmatic surface in these cases (number of flowers, and number of individuals affected, control, 15, 10; plant height reduced, 8, 6; spur length reduced, 13, 7; both traits reduced, 5, 5). As a result, the final sample sizes for analyzing effects of plant height and spur length on pollen receipt in pollinated flowers were as follows: control, 18 plants; plant height reduced, 21 plants; spur length reduced, 12 plants; and both traits reduced, 13 plants.

### 2.3 Pollinator‐mediated selection

In 2016, we quantified phenotypic selection via pollen removal and female fitness on four floral traits (plant height, number of flowers, corolla size, and spur length) in the same population. We marked about 220 plants at bolting, and once individual plants had opened at least one‐third of their flowers, floral traits were measured in the same way as described above. To separate selection through female fitness mediated by pollinators and by other selective agents, we estimated selection both among open‐pollinated control plants (net selection), and among plants receiving supplemental hand‐pollination (non‐pollinator‐mediated selection; Sandring & Ågren, 2009; Sletvold & Ågren, 2010). Two‐thirds of the marked plants were randomly assigned to the open‐pollinated control (C), and one‐third received supplemental hand‐pollination (HP). Fewer plants were assigned to the hand‐pollination treatment, because variance in female fitness is expected to be lower in this treatment compared to the open‐pollinated control. Supplemental pollinations were performed by collecting a pollinium with a toothpick from a plant not included in the experiment, or from another plant within the hand‐pollination treatment, and gently brushing it across the stigma of the receiving flower. Each plant was pollinated on three to four occasions throughout flowering, and each time, all open flowers were pollinated with pollen from a minimum of two different donors at least 5 m away from the recipient.

At the end of flowering, we recorded for each plant the total number of pollinia removed by carefully inspecting all flowers with a hand lens. At fruit maturation, we counted the number of fruits produced and harvested up to three mature fruits per plant. Fruits were brought to the lab and individually weighed to the nearest 0.01 mg as an estimate of seed production. For each plant, female fitness was quantified as the product of number of fruits and mean fruit mass.

Grazing by hare and inflorescence breakage reduced the final sample sizes to 119 plants in the open‐pollinated control and 58 plants in the hand‐pollination treatment.

### 2.4 Statistical analysis

For the analysis of the first experiment, we coded plant height as tall (treatments 1 and 2) or short (treatments 3 and 4), and spur length as long (treatments 1 and 3) or short (treatments 2 and 4). We then examined the effects of plant height (tall vs. short) and spur length (long vs. short) and their interaction on pollen removal and components of female fitness (the number of flowers receiving pollen, the mean number of massulae received by pollinated flowers, and the number of fruits produced) with mixed‐effect models, which included block as a random factor, using the lme4 package (Bates, Mächler, Bolker, & Walker, 2015) and the car package (Fox & Weisberg, 2011) to obtain type III sum of squares and *p*‐values in the software R. For significance tests, all response variables were square‐root transformed prior to analysis to obtain normality of residuals. We used posthoc Tukey test to identify statistically significant differences between treatments. To estimate least‐square means, we re‐analyzed the model using untransformed data and the package lmerTest (Kuznetsova, Brockhoff, & Christensen, 2017).

We used one‐way ANOVA to test for differences in phenotypic traits and female reproductive success between pollination treatments in the selection study. Correlations among phenotypic traits were quantified by calculating Pearson’s correlation coefficients separately by treatment. Phenotypic traits did not differ between the two pollination treatments (one‐way ANOVA, all *p* > 0.05), and trait correlations were of intermediate strength (range, 0.100–0.555; Supporting Information Table S1) with the strongest correlations recorded between plant height and number of flowers, and between flower size and spur length.

We quantified pollen removal failure in the control treatment as 1—mean proportion of pollinia removed, and pollen limitation of seed production as PL = 1—(mean female fitness in the open‐pollinated control treatment/mean female fitness in the hand‐pollination treatment).

For the analysis of phenotypic selection, we calculated relative pollen removal and relative female fitness (individual values divided by population mean values) and standardized traits separately by pollination treatment (open‐pollinated control and supplemental hand‐pollination, respectively). The opportunity for selection through pollen removal and through female fitness was quantified as the variance in relative pollen removal and relative female fitness, respectively. We used *F* test for dependent samples (Lee, 1992; Pitman, 1939) to compare the variance in relative pollen removal and relative female fitness.

We estimated phenotypic selection through pollen removal and female fitness with multiple regression models (Lande & Arnold, 1983), in which relative fitness (relative number of pollinia removed, or relative female fitness) was regressed on the four standardized traits (plant height, number of flowers, flower size, and spur length). To test for nonlinear quadratic and correlational selection, we examined models that in addition included quadratic terms and interactions among standardized traits. Quadratic selection gradients were estimated as twice the value of partial regression coefficients for quadratic terms (Stinchcombe, Agrawal, Hohenlohe, Arnold, & Blows, 2008). Selection gradients estimated in the open‐pollinated control (*β*_C_) represent total, net selection, whereas gradients estimated in the hand‐pollination treatment (*β*_HP_) represent non‐pollinator‐mediated selection. We quantified pollinator‐mediated selection through female fitness (∆*β*_Poll_) by subtracting selection gradients observed in the hand‐pollination treatment (*β*_HP_) from those observed in the open‐pollination control treatment (*β*_C_). Statistical significance of pollinator‐mediated selection through female fitness was assessed with ANCOVA models, which included the four standardized traits, and their interactions with pollination treatment (open‐pollinated control vs. supplemental hand‐pollinated) as independent variables and relative female fitness as dependent variable. A significant interaction between a given trait and pollination treatment would indicate pollinator‐mediated selection on that trait. We used the plotting and diagnostic functions of the car package within the R software (Fox & Weisberg, 2011) to check for outliers, normality and heteroscedasticity of residuals, and collinearity among variables. Collinearity was not problematic (variance inflation factors <2), and residuals were all normally distributed and homoscedastic. The shapes of fitness surfaces were explored by producing two‐dimensional added‐variable plots for quadratic selection, and by using the gam and visgam function in the package mgcv (Wood, 2017) in R to visualize three‐dimensional surfaces for correlational selection.

All statistical analyses were performed in the software R version 3.3.2 (R Developmental Core Team, 2016) using the R Studio interface.

## 3 RESULTS

### 3.1 Effects of plant height and spur length on pollen removal

**Figure 2:**
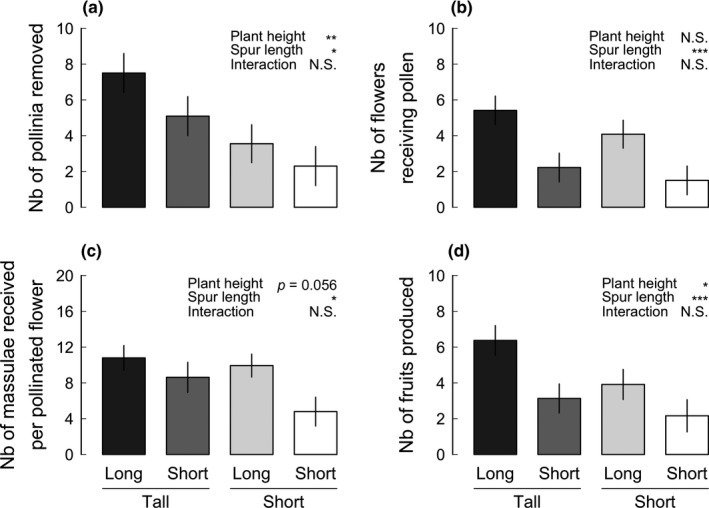
Effects of plant height (tall vs. short) and spur length (long vs. short) on (a) total number of pollinia removed, (b) the number of flowers receiving pollen, (c) number of massulae received by pollinated flowers, and (d) number of fruits produced after exposure to pollinators for three consecutive nights in a woodland population of *Platanthera bifolia* on the island Öland, SE Sweden, in 2015. Least‐square means ± *SE* extracted from model of untransformed data are given. Statistical significance of fixed factors included in mixed‐effect model is indicated (see Table 1). ***p < 0.001, **p < 0.01, *p < 0.05

Both plant height and spur length affected pollen removal (Figure 2a, Table 1). Tall plants had more than twice as many pollinia removed compared to short plants (main‐effect least‐square mean ± *SE*, 6.3 ± 0.9 vs. 2.9 ± 0.9), and long‐spurred plants had 1.5 times more pollinia removed compared to short‐spurred plants (5.5 ± 0.9 vs. 3.7 ± 0.9; Supporting Information Table S2). As predicted, the difference in pollen removal between long‐spurred and short‐spurred plants tended to be larger among tall plants (least‐square mean ± *SE*, 7.5 ± 1.1 vs. 5.1 ± 1.1 pollinia removed) than among short plants (3.6 ± 1.1 vs. 2.3 ± 1.1 pollinia removed), but the plant height × spur length interaction was far from statistically significant (*p* = 0.497, Table 1).

**Table 1:** The effect of plant height, spur length, and their interaction on number of pollinia removed, number of flowers receiving pollen, mean number of massulae received per pollinated flower, and number of fruits, analyzed with linear mixed‐effect models that included block as a random effect

Source of variation	*df*	*F*	*p*
No. of pollinia removed			
Plant height	1	9.32	**0.003**
Spur length	1	6.24	**0.015**
Plant height × spur length	1	0.47	0.497
No. of flowers receiving pollen			
Plant height	1	1.67	0.201
Spur length	1	21.36	**<0.001**
Plant height × spur length	1	0.11	0.743
No. of massulae received per pollinated flower			
Plant height	1	3.83	0.056
Spur length	1	6.62	**0.013**
Plant height × spur length	1	1.95	0.176
No. of fruits			
Plant height	1	5.26	**0.026**
Spur length	1	16.00	**<0.001**
Plant height × spur length	1	0.46	0.500

Note

Statistically significant effects (*p* < 0.05) are indicated in bold.

### 3.2 Effects of plant height and spur length on pollen receipt and fruit production

Spur length significantly affected both components of pollen receipt, whereas plant height did not affect the number of flowers receiving pollen and its effect on number of massulae received per pollinated flower was only marginally significant (Figure 2b–c, Table 1). The number of flowers receiving pollen was 2.5 times higher among plants with long spurs compared to plants with short spurs (main‐effect least‐square mean ± *SE*, 4.7 ± 0.6 vs. 1.9 ± 0.6 flowers; Supporting Information Table S2, Figure 2b), but was not significantly affected by plant height or the plant height × spur length interaction (Table 1). The number of massulae received per pollinated flower was 1.5 times higher among long‐spurred than among short‐spurred plants (10.4 ± 1.0 vs. 6.7 ± 1.2) and tended to be higher among tall than among short plants (9.7 ± 1.1 vs. 7.4 ± 1.1, *p* = 0.056; Figure 2c, Table 1); the plant height × spur length interaction was not statistically significant (*p* = 0.176, Table 1).

Long‐spurred plants produced almost twice as many fruits compared to short‐spurred plants (5.1 ± 0.7 vs. 2.6 ± 0.7 fruits; Figure 2d, Supporting Information Table S2), and tall plants produced 1.6 times more fruits than did short plants (4.8 ± 0.7 vs. 3.0 ± 0.7 fruits). As predicted, the difference in fruit production between long‐spurred and short‐spurred plants tended to be larger among tall plants (6.4 ± 0.8 fruits vs. 3.1 ± 0.8 fruits) than among short plants (3.9 ± 0.8 vs. 2.2 ± 0.9), but the plant height × spur length interaction was far from statistically significant (*p = *0.500, Table 1).

### 3.3 Pollen removal, fruit production, and opportunity for selection

In the open‐pollinated control treatment, on average 82% ± 17.9% of pollinia were removed (*N* = 118; Supporting Information Table S3) and pollen removal failure was thus 0.18. Fruit set was 75% ± 20.6% (Supporting Information Table S3), and pollen limitation of female fitness 0.13. The opportunity for selection through pollen removal was lower than that through female fitness (0.14 vs. 0.62; *F* test for dependent samples, *df* = 115, *r* = 0.558, *p* < 0.001).

### 3.4 Selection via pollen removal

There was selection for more and larger flowers, but no statistically significant selection on plant height or spur length via pollen removal (Table 2).

**Table 2:** Estimates of net selection (*β*_C_; quantified in open‐pollinated control treatment, *N* = 119), non‐pollinator‐mediated selection (*β*_HP_; quantified as selection among plants receiving supplemental hand‐pollination, *N* = 58), and pollinator‐mediated selection (∆*β*_Poll_ = *β*_C_−*β*_HP_) via pollen removal and female fitness in a woodland population of the orchid *Platanthera bifolia* on the island Öland, SE Sweden

Phenotypic trait	Pollen removal		Female fitness					
	Net selection	*p*	Net selection	*p*	Non‐pollinator‐mediated	*p*	Pollinator‐mediated	*p*
Directional selection								
Plant height	−0.024 ± 0.026	0.356	0.096 ± 0.056	0.088	−0.117 ± 0.070	0.099	**0.213 ± 0.094**	**0.024**
No. of flowers	**0.287 ± 0.023**	**<0.001**	**0.543 ± 0.050**	**<0.001**	**0.496 ± 0.065**	**<0.001**	0.048 ± 0.086	0.580
Flower size	**0.073 ± 0.025**	**0.004**	0.019 ± 0.054	0.733	0.063 ± 0.066	0.343	−0.044 ± 0.089	0.619
Spur length	−0.013 ± 0.025	0.608	0.105 ± 0.054	0.053	0.129 ± 0.071	0.074	−0.024 ± 0.093	0.800
Quadratic selection								
Plant height	0.021 ± 0.051	0.681	−0.104 ± 0.100	0.303	0.017 ± 0.181	0.925	−0.121 ± 0.218	0.580
No. of flowers	0.048 ± 0.044	0.276	**0.202 ± 0.087**	0.022	0.138 ± 0.093	0.151	0.066 ± 0.131	0.615
Flower size	−0.041 ± 0.041	0.311	0.009 ± 0.082	0.914	−0.056 ± 0.126	0.662	0.064 ± 0.157	0.683
Spur length	−0.066 ± 0.054	0.228	−0.101 ± 0.106	0.344	−0.075 ± 0.165	0.649	−0.025 ± 0.205	0.902
Correlational selection								
Plant height × no. of flowers	−0.052 ± 0.043	0.233	0.082 ± 0.086	0.339	−0.128 ± 0.127	0.318	0.210 ± 0.160	0.190
Plant height × flower size	0.032 ± 0.030	0.293	0.022 ± 0.060	0.718	−0.135 ± 0.087	0.128	0.157 ± 0.110	0.157
Plant height × spur length	−0.003 ± 0.040	0.941	0.058 ± 0.079	0.464	0.018 ± 0.099	0.854	0.040 ± 0.131	0.762
No. of flowers × flower size	**0.070 ± 0.026**	**0.010**	**−0.184 ± 0.053**	**<0.001**	0.171 ± 0.089	0.060	**−0.355 ± 0.109**	**0.001**
No. of flowers × spur length	−0.026 ± 0.025	0.303	**0.101 ± 0.050**	**0.046**	0.100 ± 0.094	0.293	0.001 ± 0.113	0.994
Flower size × spur length	0.042 ± 0.035	0.235	0.038 ± 0.072	0.597	0.139 ± 0.107	0.202	−0.101 ± 0.135	0.457

Note

Selection gradients (±*SE*) were estimated with multiple regression models, in which relative pollen removal or relative female fitness (number of fruits × mean fruit mass) were regressed on standardized traits. Statistically significant selection gradients (*p* < 0.05) are indicated in bold.

### 3.5 Selection via female fitness

**Figure 3:**
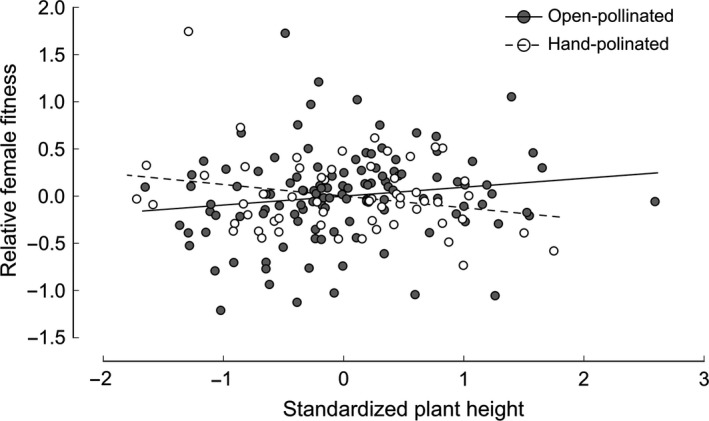
Phenotypic selection on plant height via female fitness in the open‐pollinated control treatment (filled symbols and solid line) and among plants receiving supplemental hand‐pollination (open symbols and dashed line) in a woodland population of the orchid *Platanthera bifolia* on the island Öland, SE Sweden, visualized with an added‐variable plot. In this graph, the residuals from a regression of relative fitness on all standardized traits except the focal trait is plotted against the residuals from a regression of the standardized focal trait on all other standardized traits included in the selection analysis

Among open‐pollinated plants, there was strong directional selection through female fitness for more flowers, whereas selection for taller plants and for longer spurs only approached statistical significance (Table 2, Figure 3). Relative female fitness increased disproportionately with number of flowers (significant positive quadratic selection gradient; Table 2, Supporting Information Figure S2). In addition, there was significant negative correlational selection on number of flowers × flower size, and positive correlational selection on number of flowers × spur length (Table 2, Supporting Information Figure S3) indicating selection favoring the combinations of many and small flowers, and many flowers with long spurs. However, these estimates of correlational selection were strongly influenced by a few plants with particularly high female fitness and should be interpreted with caution (Supporting Information Figure S3).

Pollinators mediated selection for taller plants (*F*_1,167_ = 8.59, *p* = 0.004, ANCOVA; Figure 3), and the negative correlational selection on number of flowers and flower size (significant treatment × number of flowers × flower size interaction, *F*_1,147_ = 13.99, *p* = 0.0003, ANCOVA; Table 2). Net selection for more flowers was mainly driven by factors other than pollinators, as indicated by the strong selection observed in the hand‐pollination treatment (Table 2).

## 4 DISCUSSION

The two ecotypes of the moth‐pollinated orchid *P. bifolia* occurring in southern Sweden differ in several traits likely to influence interactions with pollinators including plant height and nectar spur length: the woodland ecotype is taller and produces nectar spurs that are longer than those produced by the grassland ecotype. Here, we have shown with a field experiment that both differences are important for pollination success and fruit production in a woodland population. However, we did not detect any significant interaction effects of the two traits on reproductive success, and whereas pollinators mediated significant selection for taller plants, no current pollinator‐mediated selection on spur length was detected in this population. Below, we discuss the results in relation to the maintenance of the two ecotypes, and the power of experimental approaches for the identification of adaptive traits and agents of selection.

Woodland populations grow in tall vegetation and, as expected, plant height affected both pollen removal and fruit production. Reduction of plant height to that of the grassland ecotype, which grows in shorter vegetation, decreased pollen removal and fruit production, and there was pollinator‐mediated selection for taller plants. Pollinators mediate selection for taller inflorescences also in other orchid (Sletvold & Ågren, 2010; Sletvold, Grindeland, & Ågren, 2010) and non‐orchid species (Ågren, Hellström, Toräng, & Ehrlén, 2013; Paudel et al., 2016). Moreover, pollinator‐mediated selection on inflorescence height can be expected to be stronger in tall than in short vegetation (Sletvold, Grindeland, & Ågren, 2013), which is consistent with the difference in plant height between the woodland and the grassland ecotype of *P. bifolia*.

Optimal inflorescence height should depend on how this trait affects interactions also with other biotic agents and responses to the abiotic environment (Falster & Westoby, 2003). In the present study, the strong pollinator‐mediated selection for taller plants through female fitness (selection gradient Δ*β*_poll_ = 0.213) was opposed by non‐pollinator‐mediated selection for shorter plants (*β*_HP_ = −0.117) and net selection on plant height was not statistically significant. Tall plants should have an advantage over short plants when it comes to light capture in tall vegetation, but this should contribute to selection for tall rather than for short plants and can thus not explain the documented non‐pollinator‐mediated selection for shorter plants. Stronger grazing pressure on tall inflorescences may counteract the advantage of increased pollinator attraction (Ågren et al., 2013; Cariveau, Irwin, Brody, Garcia‐Mayeya, & Ohe, 2004; Gomez, 2003). However, grazed plants were not included in the selection analysis, and plant height did not differ between plants damaged by grazers and plants included in the analysis (data not shown). Additional studies are needed to test whether tall plants are more prone to experience drought stress, or whether non‐pollinator‐mediated selection for shorter plants is driven by some other biotic or abiotic environmental factors.

The length of the nectar spur should influence the morphological fit between flowers and pollinators, and therefore the efficiency of pollen transfer (Darwin, 1862; Nilsson, 1988). Here, we showed that shortening of spurs to a length corresponding to that of the grassland ecotype decreased the number of pollinia removed, the number of flowers receiving pollen, the mean amount of pollen received per flower, and fruit production in a long‐spurred woodland population of *P. bifolia*. This is in line with previous experiments conducted in the same geographic area demonstrating strong negative effects of nectar‐spur shortening on both pollen removal and receipt in the woodland ecotype (Boberg & Ågren, 2009; Nilsson, 1988). A strong effect of spur length or floral tube depth on pollen transfer has been demonstrated experimentally in several systems (e.g., Johnson & Steiner, 1997; Muchhala & Thompson, 2009; Sletvold & Ågren, 2011). Together with demonstration of pollinator‐mediated selection on spur length (e.g., Sletvold & Ågren, 2010; Sletvold et al., 2010), geographic covariation between flower tube depth and tongue length of pollinators (Anderson & Johnson, 2009; Boberg et al., 2014; Newman, Manning, & Anderson, 2014, 2015) and phylogenetic analyses of trait evolution and pollinator shifts (Koopman & Ayers, 2005; Whittall & Hodges, 2007), this suggests that pollinators have been important for the evolution of the lengths of nectar spurs and floral tubes in a wide range of species.

Although strong effects on pollen removal and fruit production were observed when spur length was reduced to a length observed in the grassland ecotype, there was no current pollinator‐mediated selection on spur length. This shows that effects on pollen removal and fruit production were weak across the phenotypic range present in the study population, and points to the general problem of determining the adaptive significance of a trait when natural variation is limited and corresponds to a flat part of the fitness landscape. Traits that have been subject to consistent stabilizing selection are expected to show limited variation (Cresswell, 1998; Fenster, 1991; Van Kleunen, Meier, Saxenhofer, & Fischer, 2008), and in this situation experimental crosses between divergent populations (Schemske & Bradshaw, 2008; Toräng et al., 2017) or phenotypic manipulation (Campbell, 2009; Peakall & Handel, 1993) may be required to characterize the relationship between trait expression and components of fitness. For example, effects of plant height on pollinator visitation in the sexually deceptive orchid *Chiloglottis trilabra* were documented only when the range of plant height was extended well beyond the natural variation (Peakall & Handel, 1993). The results illustrate that experimental manipulation may be needed for determining the adaptive significance of traits with limited variation.

Traits enhancing pollinator visitation and traits increasing efficiency of pollen transfer are expected to affect pollination success non‐additively (Benitez‐Vieyra, Medina, Glinos, & Cocucci, 2006; Engel & Irwin, 2003; Sletvold & Ågren, 2011). However, although the effect of spur length on pollen removal and female fitness tended to be stronger among tall plants than among short plants, the plant height × spur length interaction was far from statistically significant. Because the variances in both pollinia removal and fruit production were high, substantially larger sample sizes would be required for interaction effects of the estimated magnitudes to be statistically significant.

The lack of pollinator‐mediated correlational selection on plant height and spur length is not surprising given the absence of a significant plant height × spur length interaction when traits were manipulated, and the limited range in spur length in this population. A few previous studies report correlational selection supporting the hypothesis of non‐additive effects on fitness of traits important for pollinator attraction and efficiency of pollen transfer, respectively (Benitez‐Vieyra et al., 2006; Chapurlat et al., 2015; Cuartas‐Dominguez & Medel, 2010), and there was correlational selection for more flowers with longer spurs in the studied population of *P. bifolia*. However, the supplemental hand‐pollination showed that this correlational selection was not mediated by pollinators (Table 2). So far, only Chapurlat et al. (2015) have demonstrated pollinator‐mediated correlational selection on an attraction and an efficiency trait experimentally (number of flowers and spur length in the orchid *Gymnadenia conopsea*). Additional studies are required to determine whether current pollinator‐mediated selection on such traits is frequent, and whether its occurrence can be predicted based on the phenotypic variance present in a given population.

The woodland ecotype produces larger flowers than does the grassland ecotype, which may be important for its reproductive success. Flower size was not manipulated in the current study, but the phenotypic selection analysis indicated significant selection for increased flower size through pollen removal. This suggests that flower size directly, or indirectly through correlated traits, influenced either visitation rate or efficiency of pollen removal in this year. While large flowers should contribute to a prominent floral display, a previous experiment in the same woodland population found no effect of reduced perianth size on pollination success (Boberg & Ågren, 2009). This suggests that the visual display of individual flowers is not the target trait of selection. The mechanism behind the positive effect of flower size on pollen removal deserves further study.

The relative strength of selection through pollen removal and female fitness should depend on the relationship between trait expression and absolute measures of these components of fitness, the variance in trait expression, and the opportunity for selection, that is, the variance in relative fitness. Trait manipulation indicated that plant height and spur length can strongly affect both pollen removal and female fitness. However, in the natural population opportunity for selection through female fitness was markedly larger than through pollen removal, and pollinator‐mediated selection on plant height was significant only through female fitness. Still, opportunity for selection could not explain all differences in selection observed. Despite lower opportunity for selection through pollen removal, number of flowers and flower size were subject to significant selection through pollen removal, but not to pollinator‐mediated selection through female fitness indicating that variation in these traits was more important for pollen removal than for successful pollen receipt. Pollen removal has been used as a proxy for male fitness in some studies (e.g., O’Connell & Johnston, 1998; Maad, 2000; Benitez‐Vieyra et al., 2006). However, siring success depends not only on pollen removal but also on the proportion of removed pollen reaching conspecific stigmas, and the proportion of those that successfully fertilize ovules that develop into mature seeds. It is therefore not surprising that the link between pollen removal and successful pollen export to other plants (Johnson et al., 2005) and siring success (Snow & Lewis, 1993) can be weak, and additional studies are required to determine whether selection through pollen removal mirrors selection through male fitness.

Taken together, the results of the present study suggest that pollinator‐mediated selection has contributed to the divergence in both spur length and plant height between the grassland and woodland ecotype of *P. bifolia*. A history of consistent directional or stabilizing selection is expected to reduce the magnitude of trait variation in natural populations. As illustrated by this study, a combination of trait manipulation and examination of current phenotypic selection and its causes may therefore often be required for a full understanding of the functional and adaptive significance of trait divergence.

## CONFLICT OF INTEREST

None declared.

## AUTHOR CONTRIBUTION

J.T., N.S., and J.Å. planned and designed the study. J.T. performed the experiments and analyzed data, and J.T., N.S., and J.Å. wrote the paper.

## Data Availability

The data have been archived in Dryad and are accessible under https://doi.org/10.5061/dryad.6k9v66n.
