# Supplementary material for: The independent and combined effects of floral traits distinguishing two pollination ecotypes of a moth‐pollinated orchid
Source: Ecol Evol. 2019 Jan 23;9(3):1191–201. doi: 10.1002/ece3.4808 (PMC6374684; doi:10.1002/ece3.4808)

**Table S1.** Correlations among phenotypic traits in the open-pollinated control (above diagonal, *N* = 119) and among plants receiving supplemental hand-pollination (below diagonal, *N* = 58) in a woodland population of the orchid *Platanthera bifolia* on the island Öland, SE Sweden in 2016 (Pearson´s correlation coefficients). Correlation coefficients statistically significant at *P* < 0.05 are indicated in bold.

|  | Plant height | Nb flowers | Flower size | Spur length |
| --- | --- | --- | --- | --- |
| Plant height | - | **0.512** | **0.474** | **0.456** |
| Nb flowers | **0.483** | - | **0.303** | **0.260** |
| Flower size | 0.100 | 0.213 | - | **0.555** |
| Spur length | **0.401** | 0.114 | **0.501** | - |

**Table S2.** Effects of plant height and spur length on number of pollinia removed, number of flowers receiving pollen, number of massulae received per pollinated flower, and fruit production in an experiment conducted in a woodland population of *Platanthera bifolia* in 2015 on the island Öland, SE Sweden. Main-effect least square means extracted from a model of untransformed data are given. Because not all plants had information on number of massulae received per pollinated flower, sample sizes for this trait were reduced (Tall plants, *N* = 30; Short plants, *N* = 34; Long spur, *N* = 39; Short spur, *N* = 25).

| Fitness component |  | |  |  | |
| --- | --- | --- | --- | --- | --- |
|  | Tall plants (*N* = 46) | Short plants (*N* = 48) |  | Long spur (*N* = 48) | Short spur (*N* = 46) |
| Nb pollinia removed | 6.30 | 2.93 |  | 5.53 | 3.70 |
| Nb pollinated flowers | 3.81 | 2.79 |  | 4.74 | 1.86 |
| Nb massulae/poll. flower | 9.70 | 7.36 |  | 10.36 | 6.70 |
| Nb fruits | 4.75 | 3.04 |  | 5.14 | 2.64 |

**Table S3.** Phenotypic traits, and measures of pollen removal and female fitness (Mean ± SD) for open-pollinated plants and for plants receiving supplemental hand-pollination in a woodland population of the orchid *Platanthera bifolia* on the island Öland, SE Sweden, in 2016. Differences between treatments were examined with one-way ANOVA. Statistically significant differences (*P* < 0.05) are indicated in bold.

| Variable | Pollination treatment | |  |
| --- | --- | --- | --- |
|  | Open-pollinated control (*N* = 119) | Hand-pollinated plants (*N* = 58) | *P* |
|  |  |  |  |
| Plant height (cm) | 33.9 ± 6.86 | 33.2 ± 6.55 | 0.496 |
| Number of flowers | 18.4 ± 5.48 | 17.4 ± 6.31 | 0.285 |
| Flower size (cm^2^) | 3.94 ± 1.07 | 3.86 ± 0.89 | 0.658 |
| Spur length (mm) | 33.9 ± 3.68 | 33.3 ± 3.91 | 0.291 |
|  |  |  |  |
| *Pollen removal* |  |  |  |
| Number of pollinia removed | 30.3 ± 11.13 |  |  |
| Pollinia removal (%) | 81.8 ± 17.85 |  |  |
|  |  |  |  |
| *Female function* |  |  |  |
| Number of fruits | 14.2 ± 6.29 | 16.7 ± 6.02 | **< 0.010** |
| Fruit set (%) | 75.0 ± 20.57 | 96.2 ± 5.46 | **< 0.001** |
| Mean fruit mass (mg) | 16.5 ± 6.98 | 17.1 ± 6.19 | 0.536 |
| Female fitness (mg) | 258.8 ± 205.17 | 297.1 ± 188.44 | 0.233 |
|  |  |  |  |

**Figure S1**. Illustration of experimental trait manipulation and scoring of effects of pollination success in a woodland population of *Platanthera bifolia* on the island Öland, SE Sweden. (a) Reduction in spur length by carefully squeezing the nectar up, folding the tip and fixation with green elastic tape. (b) Reduction in plant height by adjusting the height of cut inflorescences mounted on a bamboo stick. (c) Visited flower with one pollinium removed.

*
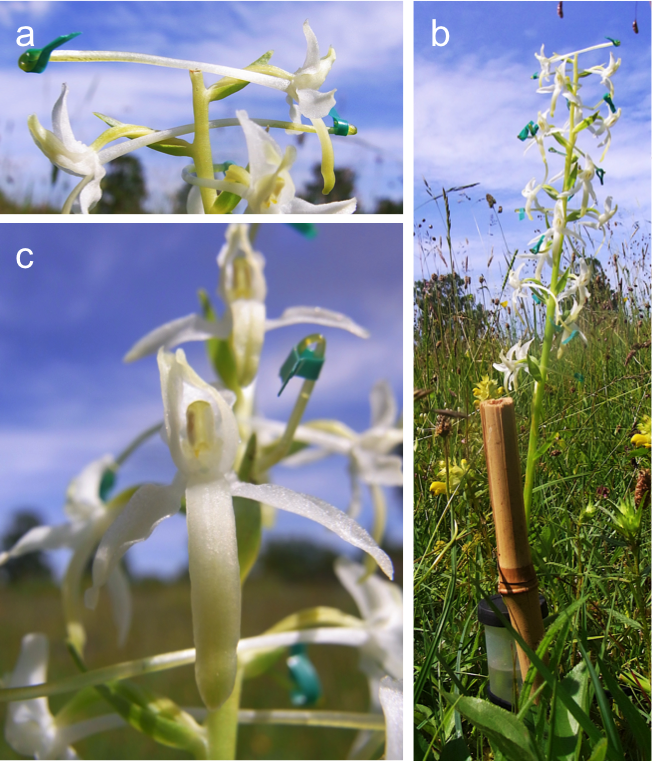
*

**Figure S2**. Added-variable plot illustrating selection on number of flowers in the open-pollinated control (net selection). The residuals of a regression of relative female fitness on the three other standardized traits included in the selection analysis are plotted against the residuals of a regression of standardized number of flowers on the three other standardized traits. A quadratic function fitted to the data is shown.


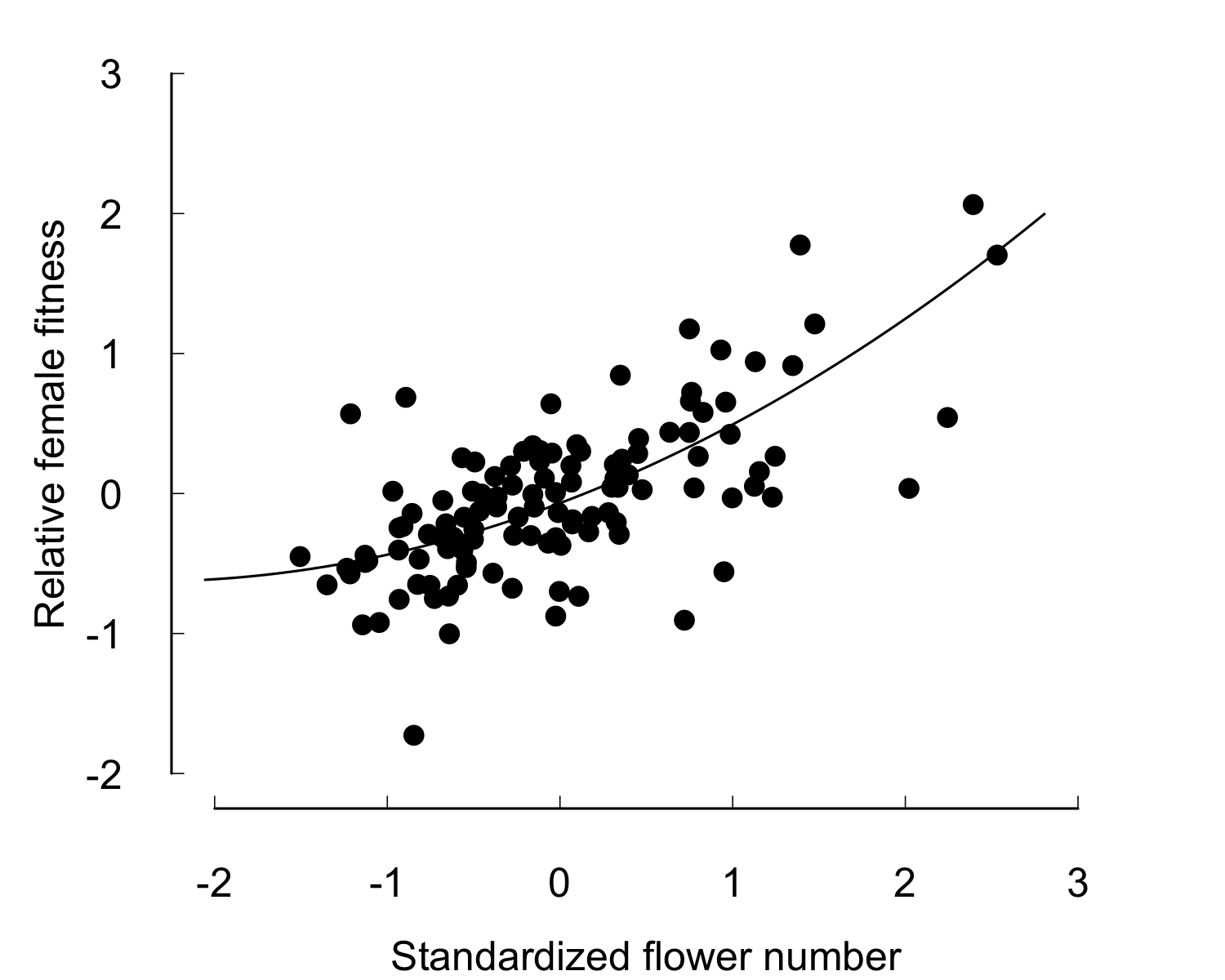


**Figure S3.** Added-variable plots illustrating correlational selection on number of flowers × flower size and number of flowers × spur length in the open-pollinated control. Plots were produced using the gam and vis.gam functions in the R package mgcv (Wood, 2017). The residuals of a linear regression of relative fitness on the two standardized traits not involved in the interaction (vertical axis) were regressed on the residuals of linear regressions of each standardized focal trait on the three other standardized traits. Fitness functions are represented as estimated 3D surfaces in the upper graphs, and as contour plots with the actual data points indicated in the lower graphs.


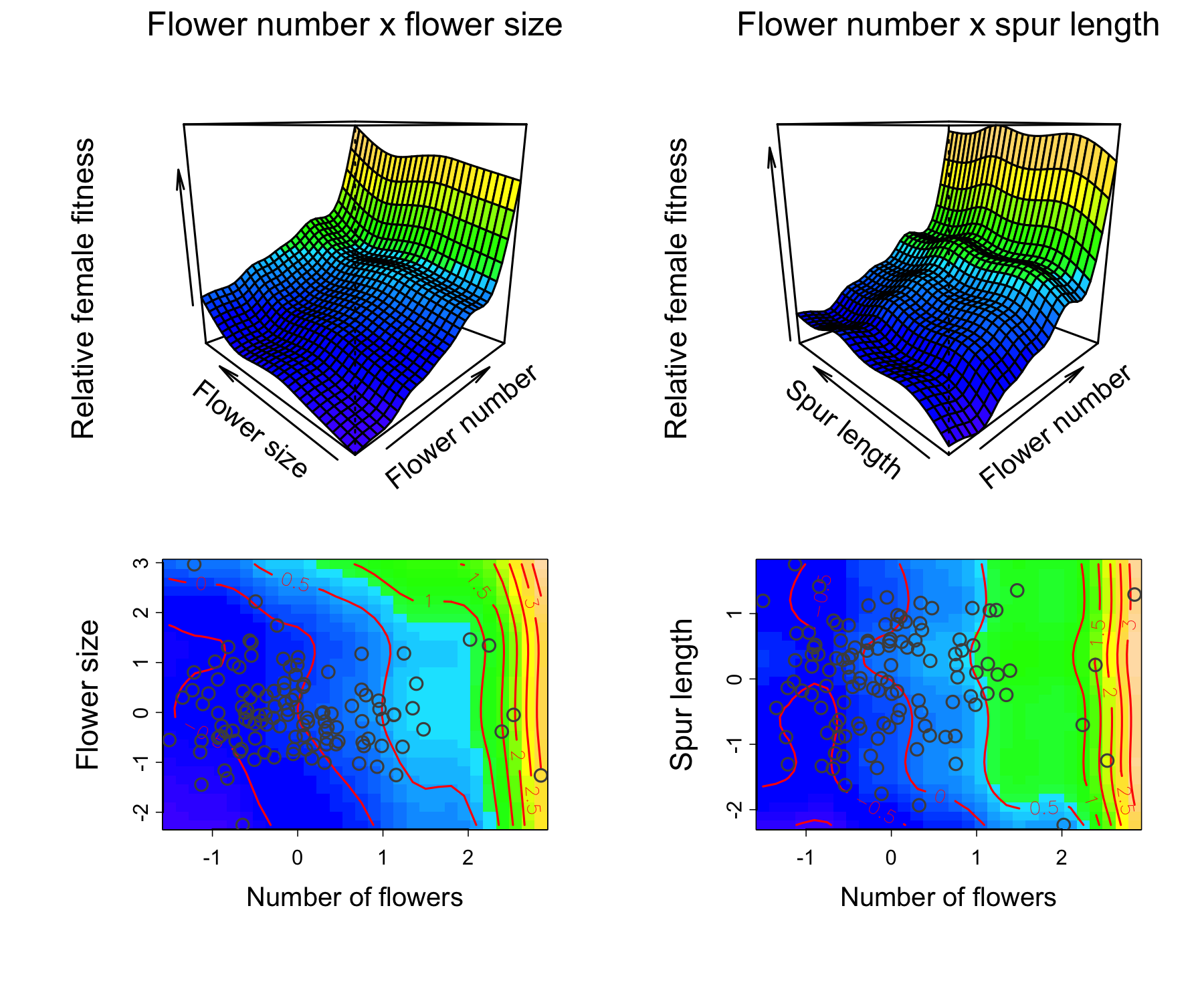

Supplement: Supplementary file 1 [file ECE3-9-1191-s001.docx]
